# Computational Docking Study of p7 Ion Channel from HCV Genotype 3 and Genotype 4 and Its Interaction with Natural Compounds

**DOI:** 10.1371/journal.pone.0126510

**Published:** 2015-06-01

**Authors:** Shilu Mathew, Kaneez Fatima, M. Qaiser Fatmi, Govindaraju Archunan, Muhammad Ilyas, Nargis Begum, Esam Azhar, Ghazi Damanhouri, Ishtiaq Qadri

**Affiliations:** 1 Department of Biotechnology, Jamal Mohamed College, Tiruchirappalli, India; 2 Center of Excellence in Genomic Medicine Research, King Abdulaziz University, Jeddah, Saudi Arabia; 3 Department of Animal Science, Bharathidasan University, Tiruchirappalli, India; 4 IQ Institute of Infection and Immunity, Lahore, Punjab, Pakistan; 5 Department of Biosciences, COMSATS Institute of Information Technology, Park Road, Chak Shahzad, Islamabad, Pakistan; 6 Department of Botany, Jamal Mohamed College, Tiruchirappalli, Tamil Nadu, India; 7 King Fahd Medical Research Center, King Abdul Aziz University, Jeddah, Saudi Arabia; University of Alabama at Birmingham, UNITED STATES

## Abstract

**Background:**

The current standard care therapy for hepatitis C virus (HCV) infection consists of two regimes, namely interferon-based and interferon-free treatments. The treatment through the combination of ribavirin and pegylated interferon is expensive, only mildly effective, and is associated with severe side effects. In 2011, two direct-acting antiviral (DAA) drugs, boceprevir and telaprevir, were licensed that have shown enhanced sustained virologic response (SVR) in phase III clinical trial, however, these interferon-free treatments are more sensitive to HCV genotype 1 infection. The variable nature of HCV, and the limited number of inhibitors developed thus aim in expanding the repertoire of available drug targets, resulting in targeting the virus assembly therapeutically.

**Aim:**

We conducted this study to predict the 3D structure of the p7 protein from the HCV genotypes 3 and 4. Approximately 63 amino acid residues encoded in HCV render this channel sensitive to inhibitors, making p7 a promising target for novel therapies. HCV p7 protein forms a small membrane known as viroporin, and is essential for effective self-assembly of large channels that conduct cation assembly and discharge infectious virion particles.

**Method:**

In this study, we screened drugs and flavonoids known to disrupt translation and production of HCV proteins, targeted against the active site of p7 residues of HCV genotype 3 (GT3) (isolatek3a) and HCV genotype 4a (GT4) (isolateED43). Furthermore, we conducted a quantitative structure–activity relationship and docking interaction study.

**Results:**

The drug NB-DNJ formed the highest number of hydrogen bond interactions with both modeled p7 proteins with high interaction energy, followed by BIT225. A flavonoid screen demonstrated that Epigallocatechin gallate (EGCG), nobiletin, and quercetin, have more binding modes in GT3 than in GT4. Thus, the predicted p7 protein molecule of HCV from GT3 and GT4 provides a general avenue to target structure-based antiviral compounds.

**Conclusions:**

We hypothesize that the inhibitors of viral p7 identified in this screen may be a new class of potent agents, but further confirmation *in vitro* and *in vivo* is essential. This structure-guided drug design for both GT3 and GT4 can lead to the identification of drug-like natural compounds, confirming p7 as a new target in the rapidly increasing era of HCV.

## Introduction

Hepatitis C virus (HCV) is chronically affecting approximately 180 million people worldwide. HCV infected individuals are at risk for liver cirrhosis as well as hepatocellular carcinoma [1, 2]. The enveloped HCV belongs to family *Flaviviridae* with seven main genotypes and roughly about 100 subtypes according to the wide geographical distribution of the HCV [3, 4]. HCV genotypes (GTs) 1–3 are distributed worldwide. The most common subtypes are 1a and 1b, accounting for about 60% of global HCV infections. These HCV subtypes prevail in Eastern Europe, Japan, and North America. GT2 remains less frequently reported than GT1. GT3 is endemic in Southeast Asia, and is unevenly distributed in various other countries around the world. GT4 is largely found in the Middle East, Central Africa, and Egypt, GT5 is almost exclusively found in South Africa, and GTs 6–11 are scattered across Asia [5–8]. The current treatment routes are limited to interferon-based and interferon-free regimens. Ribavirin and IFN-alpha-2 combination therapy has limited, but variable, effectiveness, depending on the HCV genotype and the host immune response [9, 10]. In the USA, simeprevir, an FDA approved NS3/4A protease inhibitor, is also dosed along with peg-IFN and ribavirin as triple therapy. Recently in 2011, Food and Drug Administration (FDA) and European Medicines Agency (EMEA) have approved two direct-acting antivirals (DAAs) namely boceprevir and telaprevir; these NS3/4A protease inhibitors have shown promising sustained virologic response (SVR) in phase III clinical trial, however, they are genotype specific [11]. Some combination therapies of some oral drugs have been also licensed by FDA during 2013 and 2014, which include sofosbuvir, a nucleotide analog that inhibits RNA polymerase, in combination with ribavirin for oral dual therapy of HCV GT2 and GT3 as well as sofosbuvir in combination with the viral NS5A inhibitor ledipasvir for the treatment of GT1 infection, respectively [12]. During 2012, at least 30 additional DAAs were in various stages of clinical development.

The HCV genome is expressed as large as a polyprotein and cleaved by proteases into an array of proteins. The single-stranded RNA genome encodes structural proteins, including core, glycoproteins E1 and E2, and p7, along with non-structural proteins NS2, NS3, NS4A, NS4B, NS5A, and NS5B [13]. The p7 ion channel is positioned in the middle of both the structural protein E2 and non-structural proteins [14]. HCV p7 is a viral channel-forming protein comprised of two elongated hydrophobic transmembrane (TM) domains linked by a cytosolic loop [15]. However, the structural information for p7 ion channel is known, including protein oligomerization as well as folding of the helices [16, 17]. The hexameric bundle structure was reported for the first time in a Nuclear Magnetic Resonance (NMR) spectroscopic study; the three-dimensional structure of the hexamer was generated using computational methods [18]. The recent advances in computational techniques have enabled us to build small protein molecules and portions of larger protein molecules with reasonably good resolution. Various approaches have been developed and adopted, including a combination of modeling, molecular docking, and molecular dynamics simulations [19]. Computer-modeling of proteins is guided by the knowledge of how membrane proteins are folded or inserted into the lipid membrane. Membrane proteins are translated with the aid of translocons [20–22]. Translocons are membrane-spanning proteins that enable the primary sequence of the membrane protein to form secondary structure within the hydrophobic region of the lipid membrane. The final topology of the membrane protein is dictated by the prime amino acid sequence of the protein [23–25]. The protein is finally released into lipid bilayer. Thus, once the secondary structure is formed, the protein retains this folded structure. These viral channel-forming proteins can also be built alone using computational techniques [26, 27].

Apart from forming a self-assembled, sophisticated, funnel-like architecture that selectively conducts cations, the p7 protein also plays a crucial role in viral assembly and envelopment processes in coordination with NS2 protein [14, 28, 29]. Steinmann and co-workers have shown that the production of viral particle by p7 is genotype-specific due to its interaction with other viral factors. However, the interaction pattern of p7 protein with other viral and host factors as well as its exact contribution in viral production remains uncertain [29]. Recently, a co-immunoprecipitation study using a replication-competent virus containing a double HA-tagged p7 was performed by Vieyres and co-workers that endorsed the formation of specific interaction between p7 and NS2, and highlighted its importance in virus production in cell culture [30]. Although the basic fundamental structures of p7 are becoming gradually understood, the conditions that lead to disruption of the assembly of the functional channel as well as the mechanism of drug-interactions are unknown. Furthermore, absence of clinical efficiency with current p7 inhibitors has cast doubt over their precise antiviral effects. Adamantine, rimantadine and alkylated imino sugars (IS) are known and identified as having particular resistance mutations that ascribe their methods of inhibition. Sensitivity of p7 ion channel activity to inhibition has been reported *in vitro* with hexamethylene chloride, adamantine, as well as long chain imino sugars. These inhibitors are active against only certain HCV genotypes, and various groups have reported differing sensitivities [16]. Present interferon-based therapy for HCV infected patients is insufficient, stimulating a route for combination of direct-acting antiviral (DAA). Several compounds targeting the three non-structural viral proteins NS3/4A protease, NS5A, and NS5B polymerase, still must be assessed.

p7 oligomerizes in the phospholipids membranes forming a cation-selective ion channel [31, 32], which is known for drug targetable region for molecular activity of protein; that is so far characterized. p7 is also grouped to the family of viroporin as the HIV-1 Vpu and influenza A protein M2 [15, 33, 34]. Viroporin inhibitors such as rimantidine and amantadine were first approved over 40 years ago as anti-influenza A drugs, proving an effective pharmacological expansion in the class of anti-viral complexes [35]. Rimantidine and amantadine hinder influenza A by obstructing H+ conduction via the M2 ion channel, thereby disturbing the conformational change in the viral proteins required for viral replication [36]. Recently, it was shown that p7 mediates cation conductance, and is inhibited by adamantine, long alkyl chain imino sugar and amiloride *in vitro* with varying reported efficacies [31, 37–39]. Additionally, p7 is known to precisely interact with the non-structural NS2 protein, indicating that its channel activity can be regulated [30, 40].

An *in silico* approach combining global search engines and macromolecule-ligand computational docking was applied to create the best possible models for assembled p7. We compared the inhibitory efficacy of three reported p7 inhibitors. In the study presented here, we modeled the monomeric p7 structures from GT3 (Asia) and GT4 (Middle East) to develop 3D structures, which were evaluated by protein simulation and PROCHECK. In addition, we docked the selected ligands into active site regions of the modeled structure from both genotypes. We focused on constructing and evaluating the 3D structures from representative with the type of interaction made with the residues of the model. Our results reveal a possible role for residues interacting with the p7 ion channel. It is likely that the future inhibitor natural compounds against p7 will have to be tested on multiple genotypes to determine the potential clinical efficacy.

## Materials and Methods

The sequences for p7 ion channel, each consisting of 63 amino acids, were retrieved from UniProtKB. It was ensured that all the selected viral strains use homo sapiens as their host (Table 1). The UniProtKB entries for 12 strains of HCV GT1 are P27958, P26664, Q00269, Q9WMX2, Q03463, P26662, Q913V3, O92972, P26663, P29846, Q81754 and Q913D4; for HCV GT2 (6 strains) are P26660, Q99IB8, P26661, Q9DHD6, Q68749 and Q9QAX1; for HCV GT3 (4 strains) are Q81495, Q81258, Q81487 and Q68801; for GT4 (7 strains) are O39929, **M1VKT9, A2CJ00, Q1ZZ56, A0A023JCC8**, **A8S500 and A8S507;** for GT5 (2 strains) are O39928 and O91936; and for HCV GT6 (5 strains) are Q5I2N3, O39927, O92529, O92530 and O92532.

**Table 1:** The table denotes 63 amino acid residues of p7 ion channel obtained from complete HCV genome of various HCV genotypes and subtypes isolated from different countries.

Entry code	Isolated	Virus host	Chain	amino acid	HCV p7 (all genotypes)	strains	Subtypes
P27958	North America	Homo sapiens (Human)	747–809	63	>isolate H-1a|P27958|747-809ALENLVILNAASLAGTHGLVSFLVFFCFAWYLKGRWVPGAVYALYGMWPLLLLLLALPQRAYA	isolate H	1a
P26664	North America	Homo sapiens (Human)	747–809	63	>isolate1-1a|747-809ALENLVILNAASLAGTHGLVSFLVFFCFAWYLKGKWVPGAVYTFYGMWPLLLLLLALPQRAYA	isolate 1	1a
Q00269	Japan	Homo sapiens (Human)	747–809	63	>isolate HC-JT-1b|Q00269|747-809ALENLVVLNAASLAGADGILSFLVFFCAAWYIKGRLVPGAAYALYGVWPLLLLLLALPPRAYA	isolate HC-JT	1b
Q9WMX2	Europe	Homo sapiens (Human)	747–809	63	>isolate Con1-1b|Q9WMX2|747-809ALENLVVLNAASVAGAHGILSFLVFFCAAWYIKGRLVPGAAYALYGVWPLLLLLLALPPRAYA	isolate Con1	1b
Q03463	Japan	Homo sapiens (Human)	747–809	63	>isolate HC-J1-1bALENLVILNAASLAGTRGLVSFLVFFCFAWYLKGRWVPGAAYALYGMWPLLLLLLALPQRAYA	isolate HC-J1	1b
**P26662**	Japan	Homo sapiens (Human)	747–809	63	>isolate Japanese-1b|P26662|747-809TLENLVVLNAASVAGAHGLLSFLVFFCAAWYIKGRLVPGAAYALYGVWPLLLLLLALPPRAYA	isolate Japanese	1b
Q913V3	Europe	Homo sapiens (Human)	747–809	63	>isolate HCR6-1b|747-809ALENLVVLNAASVAGAHGILSFLVFFCAAWYIKGKLVPGAAYAFYGVWPLLLLLLALPPRAYA	isolate HCR6	1b
O92972	Japan	Homo sapiens (Human)	747–809	63	>strain HC-J4-1b|O92972|747-809ALENLVVLNAASVAGAHGILSFLVFFCAAWYIKGRLAPGAAYAFYGVWPLLLLLLALPPRAYA	strain HC-J4	1b
P26663	Asia	Homo sapiens (Human)	747–809	63	>isolate BK-1b|P26663|747-809ALENLVVLNSASVAGAHGILSFLVFFCAAWYIKGRLVPGATYALYGVWPLLLLLLALPPRAYA	isolate BK	1b
P29846	Taiwan	Homo sapiens (Human)	747–809	63	>isolate Taiwan-1b|P29846|747-809ALENLVVFNAASVAGMHGTLSFLVFFCAAWYIKGRLVPGAAYALYGVWPLLLLLLALPPRAYA	isolate Taiwan	1b
P26660	Japan	Homo sapiens (Human)	751–813	63	>isolateHC-J6-2a|P26660|751-813ALEKLVVLHAASAASCNGFLYFVIFFVAAWYIKGRVVPLATYSLTGLWSFGLLLLALPQQAYA	isolate HC-G9	2a
Q99IB8	India	Homo sapiens (Human)	751–813	63	>isolateJFH-1-2a|Q99IB8|751-813ALEKLVVLHAASAANCHGLLYFAIFFVAAWHIRGRVVPLTTYCLTGLWPFCLLLMALPRQAYA	isolate India	2a
P26661	Japan	Homo sapiens (Human)	751–813	63	>isolateHC-J8-2b|P26661|751-813ALEKLIILHSASAASANGPLWFFIFFTAAWYLKGRVVPVATYSVLGLWSFLLLVLALPQQAYA	isolate HC-J6	2b
Q9DHD6	POLG_HCVJP	Homo sapiens (Human)	751–813	63	>isolate JPUT971017-2b|Q9DHD6|751-813ALEKLIILHSASAASANGPLWFFIFFTAAWYLKGRVVPAATYSVLGLWSFLLLVLALPQQAYA	isolate JFH-1)	2b
Q68749	POLG_HCVBB	Homo sapiens (Human)	751–813	63	>isolateBEBE1-2c|Q68749|751-813ALEKLVILHAASAASSNGLLYFILFFVAAWCIKGRAVPMVTYTLLGCWSFVLLLMALPHQAYA	isolate HC-J8	2c
Q9QAX1	POLG_HCVVA	Homo sapiens (Human)	751–813	63	>isolateVAT96-2k|Q9QAX1|751-813ALEKLVILHAASAASSHGMLCFIIFFIAAWYIKGRVTPLVTYSYLGMWSFSLLLLALPQQAYA	isolate JPUT971017	2k
Q81487	POLG_HCVTR	Homo sapiens (Human)	755–817	63	>isolateTr-Kj-3b|Q81487|755-817AMENLVMLNALSAAGQQGYVWYLVAFCAAWHIRGKLVPLITYGLTGLWPLALLDLLLPQRAYA	isolate BEBE1	3b
Q68801	POLG_HCVJK	Homo sapiens (Human)	751–813	63	>isolateJK049-3k|Q68801|752-814ALENLIVLNAISAAGTHGIWWSLVAFCVAWHVRGRIFPIAVYSIVGLWPLLLLVLMLPYRAYA	isolate VAT96	3k
O39929	POLG_HCVED	Homo sapiens (Human)	747–809	63	>isolateED43-4a|O39929|747-809ALSNLININAASAAGAQGFWYAILFICIVWHVKGRFPAAAAYAACGLWPCFLLLLMLPERAYA	isolate k3a	4a
M1VKT9	M1VKT9_9HEPC	Homo sapiens (Human)	747–809	63	>isolateM1VKT9-4a|747-809ALSNLININAASAAGTQSFWYAILFICIAWHVKGRLPAIAAYAACGMWPLLLLLLMLPERAYA	isolateM1VKT9	4a
A2CJ00	A2CJ00_9HEPC	Homo sapiens (Human)	747–809	63	>isolateA2CJ00-4d|747-809LANLITINAVSVAGIHGFWHAILLICIAWHVKGRFPAAATYAACGLWPLLLLVLMLPERAYAF	isolateA2CJ00	4d
Q1ZZ56	Q1ZZ56_9HEPC	Homo sapiens (Human)	747–809	63	>isolateQ1ZZ56-4d|747-809LANLVTINAVSAAGTHGFWYAILVICIAWHVKGRIPAAATYAACGMWPLLLLVLMLPERAYAF	isolateQ1ZZ56	4d
A0A023JCC8	A0A023JCC8_9HEPC	Homo sapiens (Human)	747–809	63	>isolateA0A023JCC8-4d|747-809LANLITINAVSVASIHGFWYAIFVICIAWHVKGKLPAAATYAACGLWPLLLLVLMLPERAYAF	isolateA0A023JCC8	4d
A8S500	A8S500_9HEPC	Homo sapiens (Human)	747–809	63	>isolateA8S500-4f|747-809EAALTNLININAAAAVGTHGFYYAILFICVVWYIKGRAPAAAAYAACGMWPLLLLLLALPERA	isolateA8S500	4f
A8S507	A8S507_9HEPC	Homo sapiens (Human)	747–809	63	>isolateA8S507-4f|747-809AALANLITINATAAVGTHGFCYAILFICVVWYIKGRGPAAAAYAACGMWPLLLLLLALPERAY	isolateA8S507	4f
O39928	New Zealand	Homo sapiens (Human)	748–810	63	>isolateEUH1480-5a|O39928|748-810TCKNVIVLNAAAAAGNHGFFWGLLVVCLAWHVKGRLVPGATYLCLGVWPLLLVRLLRPHRALA	isolate NZL1	5a
O91936	POLG_HCVSA	Homo sapiens (Human)	748–810	63	>isolateSA13-5a|O91936|748-810ALENVIVLNAAAAAGTHGFFWGLLVICFAWHFKGRLVPGATYLCLGIWPLLLLLFLLPQRALA	isolate Tr-Kj	5b
Q5I2N3	POLG_HCV6A	Homo sapiens (Human)	752–814	63	>isolate6a33-6a|Q5I2N3|752-814AVERLVVLNAASAAGTAGWWWAVLFLCCVWYVKGRLVPACTYMALGMWPLLLTILALPHRAYA	isolate JK049	6a
O39927	POLG_HCVEU	Homo sapiens (Human)	751–813	63	>isolateEUHK2-6a|O39927|751-813AVERLVVLNAASAAGTAGWWWAVLFLCCVWYVKGRLVPACTYMALGMWPLLLTILALPPRAYA	isolate ED43	6a
O92529	POLG_HCVT5	Homo sapiens (Human)	752–814	63	>isolateTh580-6b|O92529|752-814ALERLVVLNAASAAGTAGWCWTLIFLCCVWHVKGRLVPACTYTALGMWPILLVILALPQRAYA	isolate EUH1480	6b
O92532	POLG_HCVVP	Homo sapiens (Human)	752–814	63	>sp|O92532|748-810ALENVIVLNAASAASCQGLLWGLIFICCAWHVRGRAVPVTTYALLQLWPLLLLILALPRRAYA	isolate VN004	6h
O92531	POLG_HCVVO	Homo sapiens (Human)	752–814	63	>sp|O92531|749-811ALENLIVLNATSAAGSQGWVWGVVFICAAWYIRGRAAPITTYAILQLWPLLLLVLALPRRAYA	isolate VN405	6k

Each strain is represented by Uniprot with their entry code.

### PRALINE multiple sequence alignment

Multiple sequence alignment was done on the FASTA format of p7 ion channel for all genotypes by using IBIVU server [41–43]. PRALINE multiple sequence alignment used BLOSUM62 weight matrix algorithm with gap penalty and extension values of 12 and 1, respectively. PSI-BLAST pre-profile processing (Homology-extended alignment) was used for progressive alignment strategy [44, 45]. The alignment was also based on structural features which used DSSP-defined secondary structure using PSIPRED method [41].

### Phylogenetic analysis

The Phylogeny.fr platform [46] was used to generate phylogenetic tree of all HCV genotypes in order to find out the evolutionary relationship among them. The processing steps of multiple alignment and refinement were done by using programs called MUSCLE 3.7 and Gblocks 0.91b, respectively. The parameter values of 16, 26 and 8 were used for minimum number of sequences for conserved position, minimum number of sequences for flanking position and maximum number of contiguous non conserved positions, respectively. Phylogenetic tree was constructed and visualized by PhyML 3.0 and TreeDyn 198.3 programs, respectively. Branch support (displayed in % and colored in red) was estimated with the approximate likelihood ratio test (aLRT) method as implemented in PhyML 3.0.

### Sequence alignment and homology modeling

The protein sequence of HCV p7 ion channel, retrieved from SWISS PROT database, contains about 63 amino acid residues [47]. ClustalW was used for multiple sequence alignment of protein FASTA sequence between prevalent HCV GT3 [48] and HCV GT4 (subtype-ED43) in Asia and Middle East. Multiple alignment parameters includes weight matrix that uses BLOSUM with gap open penalty and gap extension penalty values of 10 and 0.05, respectively [49] in Asia and Middle East. Multiple alignment parameters includes weight matrix that uses BLOSUM with gap open penalty and gap extension penalty values of 10 and 0.05, respectively [50].

The crystal structure of hepatitis C virus of GT1 [51] (PDB entry code3ZDO) was identified as a homologous protein of p7 domains, 753–815 and 747–809, of GT3a (Q81495) and GT4a (O39929), respectively, by using BLAST against PDB database. The crystal structure was then used as a template to model p7 ion channel of HCV GT3 and GT4. The energy minimization of the modeled proteins was done by using ModRefiner [52], which follows two-step procedure for constructing full-atom model. The first step builds the backbone for the available C-alpha and performs energy minimization to improve the quality followed by the second step which adds side chain atoms from a rotamer library, and conducts energy minimization to both side chains and backbone conformations [52].

### 3D structure validation

The final refined p7 models for HCV GT3 and GT4 were validated by using PROCHECK (Structural Analysis and Verification Server) to calculate the Ramachandran plot [53]. SuperPose version 1.0 was used to analyze energy criteria of modeled proteins for genotype 3 and 4 with 3D template structure [54], and to calculate the root mean square deviation (RMSD) value with the template [54].

### Ligand generation and optimization

The structure of the four available drug molecules such as, long-alkyl-chain iminosugar derivatives (N-Butyldeoxynojirimycin NB-DNJ), hexamethylene amiloride, amantadine and BIT225 were obtained from PubChem [55] Similarly the 2D chemical structure of the natural molecules which has antiviral properties, were also drawn by using ACD Chemsketch. CHARMM force field was applied for energy minimization to obtain a convergence gradient by using CHARMM Boundary Potential Builder [56]. (Represented in Table 2.)

**Table 2:** Different types of active ligands taken into *in silico* study, with their chemical formula and their predicted function in disrupting different stages of viral cycles.

S.No	Drug names	Chemical Formula	Viral step	References
1	Amantadine	C10H17N	Ion channels activities	[74]
2	Hexamethylene Chloride	C12H30Cl2N2	HCV particle production	[31, 74, 85]
3	NB-DNJ	C10H21NO4	Folding of viral envelope and production of viral particles	[29, 39, 78]
4	BIT225	C16H15N5O	Ion channels activities	[80]
5	Apigenin	C15H10O5	HCV infection	[74, 86]
6	EGCG	C22H18O11	Early step of entry glycoproteins (attachment)Cell-to-cell spreadClearance of cell culture	[64, 87]
7	Ladanein	C17H14O6	HCV entry	[88]
8	Luteolin	C21H20O11	Replication	[86]
9	Naringenin	C15H12O5	AssemblySecretion (core and HCV RNA)	[89, 90]
10	Quercetin	C15H10O7	HCV replicationHCV production	[91, 92]
11	Silymarin	C25H22O10	Entry (fusion)ReplicationRNA and protein expression	[37, 93, 94]
12	Honokiol	C18H18O2	EntryReplication	[95]
13	Nobelitin	C21H22O8	Replication	[96, 97]

### VEGA-QSAR

A virtual model for property evaluation of chemicals within global architecture-quantitative structure-activity relationship (VEGA-QSAR) program was used to analyze the selected ligands to determine the relationship of physiochemical properties and biological activities of descriptor molecules in various classified QSAR models. QSAR models initially summarize a theoretical relationship between chemical structures and biological activity in a data-set of chemicals. Secondly, QSAR models determine the activities of new chemical compounds. Toxicity, ecotoxicity, predicted physiochemical properties of ligands such as logP (CAESAR-version 1.1.2), bio concentration factor (BCF) (CAESAR-version 2.1.13), carcinogenicity model (CAESAR 2.1.8), mutagenicity model (CAESAR version 2.1.12), skin sensitization model (CAESAR-version 2.1.5), developmental toxicity model (CAESAR-version 2.1.6), fathead minnow LC50 96hr (lethal concentration to kill 50% of the test animals) (Environmental Protection Agency (EPA)-version1.0.6), daphnia magna LC50 48hr (EPA-version 1.0.6), BCF reads across model (version-1.0.2), and ready biodegradability model (version 1.0.8) were determined. The VEGA-QSAR models were initially derived from CAESAR models, and other models were added to stimulate the already available models, one such model is EPA (US Environmental Protection Agency). The used input formats were SMILES and SDF files [57].

### Docking studies

Comparative molecular docking study between HCV p7 GT3 and p7 GT4 with the selected molecules was performed by using CLC drug discovery workbench, which follows a template docking algorithm, and uses MolDock scoring function for binding energy calculations [58]. Molecule project system is initially used to upload the PDB files of the modeled p7 structures, containing a binding site setup as input. For each of the small molecules in the molecule table, the docking simulation searches for optimal binding modes to the binding site. A maximum of 10 binding modes of ligands for each p7 protein were generated by using default parameter of CLC drug discovery. The docking score used in the Drug Discovery Workbench is the PLANTS_PLP_ score [59]. This score has a good balance between accuracy and evaluation time. The score mimics the potential energy change, when the protein and ligand come together. This means that a very negative score corresponds to a strong binding and a less negative or even positive score corresponds to a weak or non-existing binding. Based on the number of HBond interactions and docking score, the best-ranked compounds were selected for detailed binding interaction studies. The ligand-protein complexes were visualized in CLC drug discovery visualization tool [60].

## Results and Discussion

### Phylogenetic analysis

**Fig 1:**
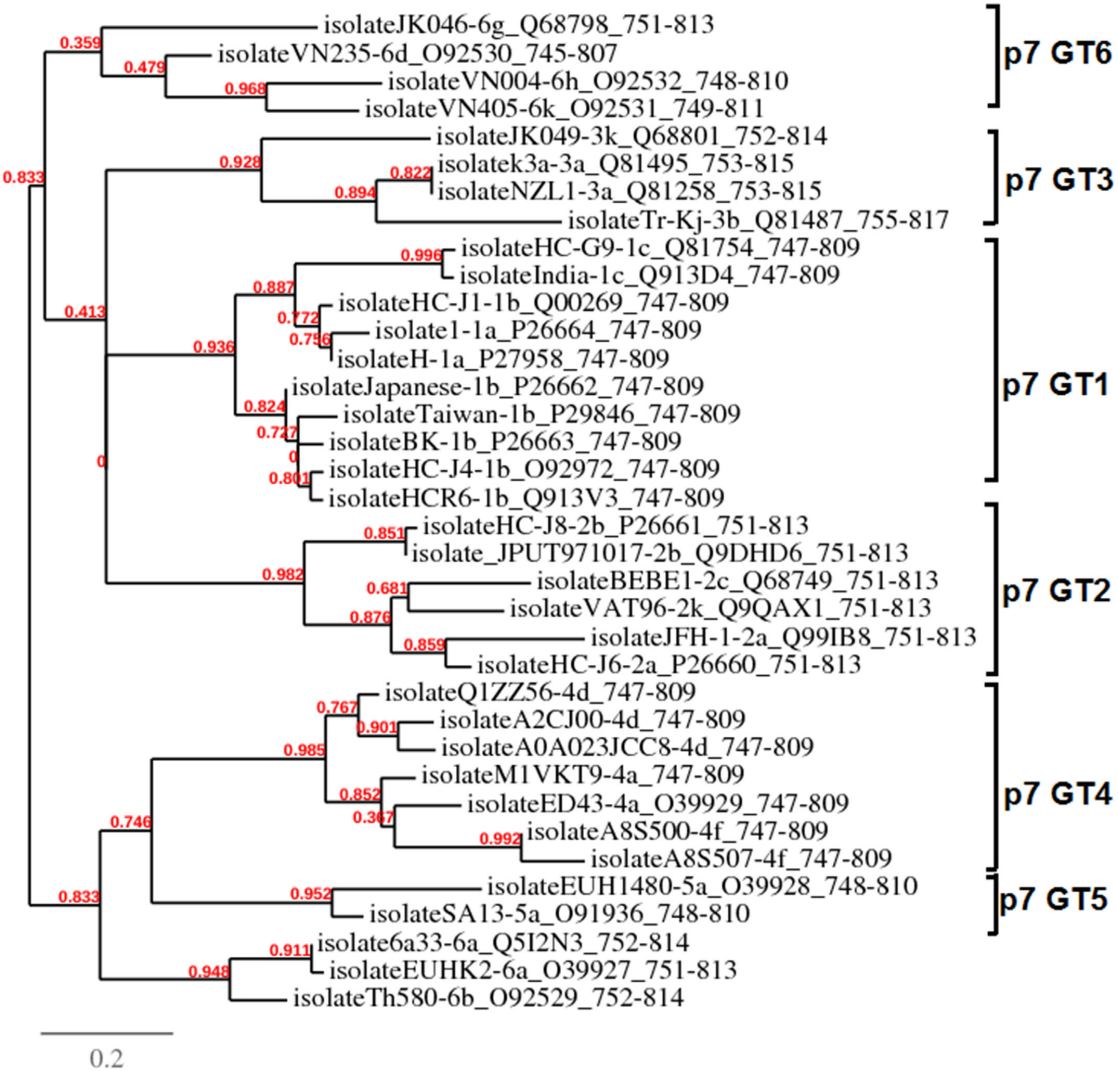
Phylogenetic relationships among different HCV genotypes and their subtypes. The numbers (shown in red) above the branches are the maximum likelihood branch support values that are obtained by using PhyML 3.0 program.

The amino acid sequences of the p7 ion channel from all complete HCV genotypes were aligned to generate a maximum likelihood tree (PhyML) with a divergent outgroup between each subtypes. The results of phylogenetic analyses are summarized in Fig 1. The p7 ion channel of GT1 shows closer similarity within different strains of subtype b. Strains from subtype a (GT1) are similar to GT3 p7 subtypes. p7 from subtypes GT4 and GT5 are predicted to have maximum likeness. **Strains from GT4 subtype 4d such as isolateQIZZ56, isolateA2CJ00 and isolateA0A023JCC8 formed a cluster indicating low sequence variation as compared to those from GT4 subtypes 4f such as isolateA8S500 and isolateA8S507 which showed maximum similarity.** The GT6 is slightly distributed between GT4, GT5, and GT2. The branch leading to GT4a isolateED43 is long, potentially because it had the most time to evolve. The rooting of the GT1 clade is less divergent among its subtypes compared to the vast divergence between GT4, GT5, and GT6. At the base of the tree, HCV GT1 subtypes 1a and 1b are clustered and diverse from the global subtype 1c, whereas both GT2 and GT3 subtypes are clustered together in their respective clade. Among the GT2 phylogenies, the 2a, 2c, and 2k subtypes formed a cluster diverse from the 2b subtypes. Within the core phylogeny, only strain isolateHC-J8-2b is present separate from this cluster, possibly due to less bootstrap support arised from inadequate phylogenetic details. Similarly, strain (isolate6a33) clustered outside, compared to subtype 6a (isolateEUHK2) and 6b (isolateTh580). GT6 subtypes are genetically very diverse, are distributed throughout the tree, and tended to be found at the base of the HCV GT4 and GT5 branches. Amino acid substitution/mutation rate per site in p7 protein among all genotypes of HCV was found to be 0.03%, meaning that the virus has accumulated a significant number of substitutions. Thereby, the phylogenetic analysis of HCV p7 genotype isolates from different strains and reference strains from various other parts of the world divulges great genomic diversity of GT4, GT5, and GT6, less diversity of GT1, and moderate diversity in GT2 and GT3.

### Consensus sequence alignment

**Fig 2:**
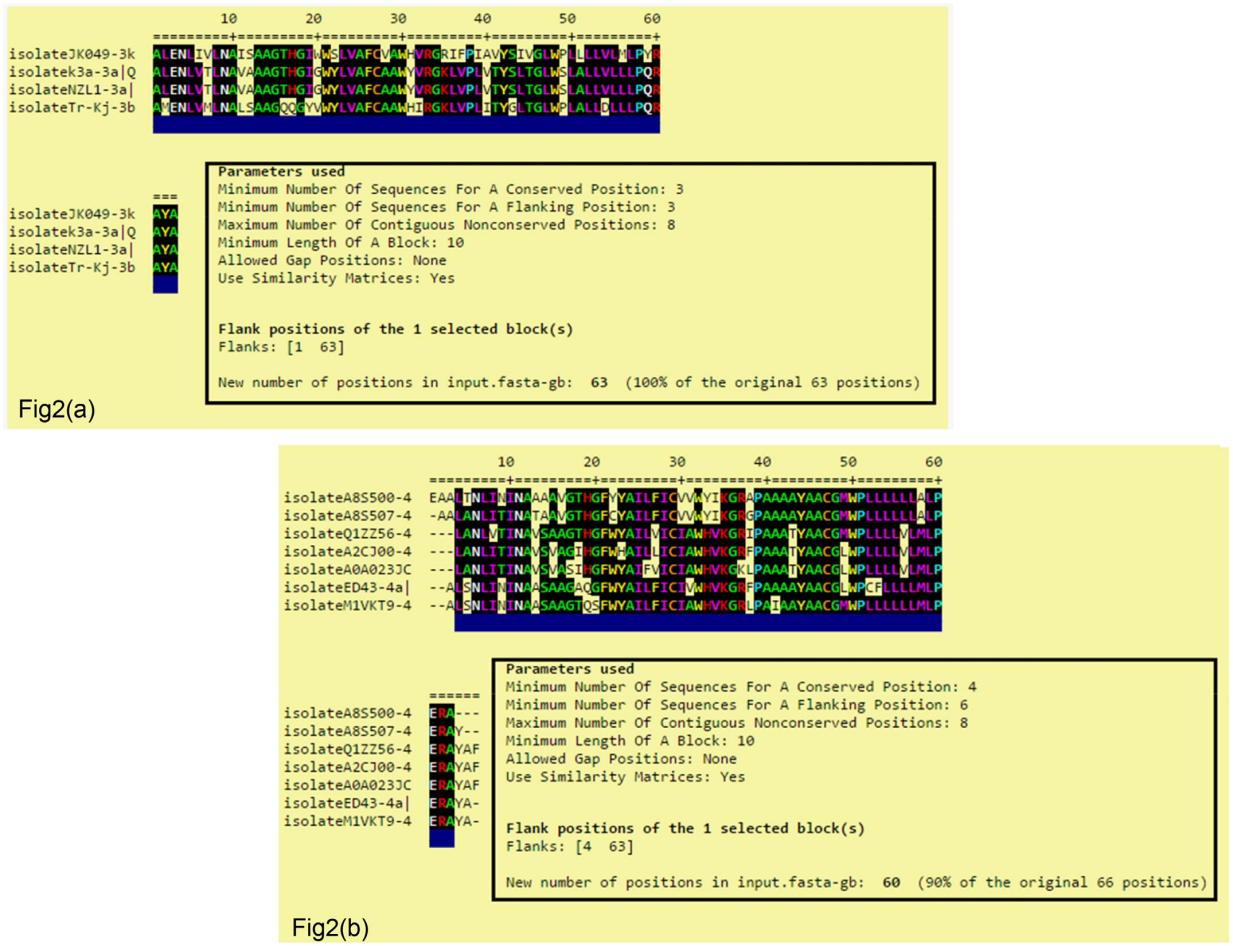
Multiple sequence alignment of HCV p7Comparative sequence alignment of HCV p7 with all GT3 subtypes using ClustalW program. The conserved residues in all sequences have been highlighted by black color, as it denotes residues sharing ‘very similar’ and ‘less similar’ properties at that position, respectively. If there are no highlights, it denotes that there is no common residue in that position of the sequence. Isolatek3a and isolateNZL1 from GT3a showed maximum similarity compared to strains isolateJK049 and isolateTr-Kj. Sequence alignment was also performed within GT4 subtypes using ClustalW tool. The conserved regions sharing very similar sequence are highlighted in black. The GT4 subtypes showed much variation in the N-terminal region (1-13aa), followed by loop region (25-45aa) compared to C-terminal region (50-63aa).

The multiple sequence alignment of the consensus sequence from both GT3 and GT4 isolates studied is shown in Fig 2a & 2b. The exact HCV subtype 3a of GT3 represents the subtype 3a strain from Asia (isolatek3a), and has 100% identity with the New Zealand strain (isolateNZL1). The percent sequence identity among different subtypes of GT3 varied between 62% and 78%. Similarly, the consensus sequence alignment of HCV subtype 4a of GT4 was also performed, which showed a maximum identity of 87% with isolateED43 and isolateM1VKT9, whereas the percent sequence identity among the different subtypes of GT4 such as isolateA8S507, isolateA8S500, isolateA0A023JCC8, isolateQ1ZZ56, and isolateA2CJ00 varied between 73% and 77%. We observed less variation in residues at the C-terminal region of p7 as compared to the loop region and N-terminal region in GT4 sequences but each subtype of GT4 was consistently mutated at 39 positions which could be considered as novel, as the amino acid residue varied at that particular position for each GT4 sequence. Comparative sequence alignment was also performed separately for both GT3 p7 subtypes as well as GT4 p7 subtypes to identify the sequence with maximum conserved residues so that the target region for interaction of protein residues with the ligand molecules can be defined. Therefore, subtype 3a (isolatek3a) from GT3 and subtype 4a (isolateED43) from GT4 were further taken for homology modeling, as they showed maximum similarity with their respective genotypes compared to other subtypes. Multiple sequence alignment of protein FASTA sequence for the complete set of p7 sequences from all genotypes was also analyzed to highlight the conserved and mutated region which is presented in S1 Dataset.

### BLAST alignment

We queried the reference sequence of the HCV GT3 p7 and HCV GT4 p7 target sequences by using the BLASTp (protein basic local alignment search tool) search. The BLASTp search revealed several sequences homologous to ion channel p7 GT3a (isolatek3a) and GT4a (isolateED43); the measles virus phosphoprotein (PDB code 3ZDO), was chosen as the best template for modeling the GT3 and GT4 p7 models. The 3ZDO ‘A’ chain, which had maximum identity with both of the selected genotypes, was chosen as a template sequence. The atomic structure of the stable domain of the measles virus phosphoprotein has a tight, four-stranded coiled coil, and consists of chains A, B, C, D, E, F, G, and H. This crystal structure was determined using X-ray diffraction at a resolution of 2.07 Å and was observed with more than one probable quaternary state. 3ZDO was obtained from the tetramerization domain of measles virus phosphoproteins, and had 56% identity to both the targets with query coverage of 100%. Accordingly, we generated a 3D macromolecule of 63 amino acid residues of the target GT3a and GT4a p7, based on alignment and modeling from an 84 amino acids sequence from chain A (3ZDO) of the measles virus phosphoprotein by a homology modeling procedure.

### Comparison of Ramachandran plots

**Fig 3:**
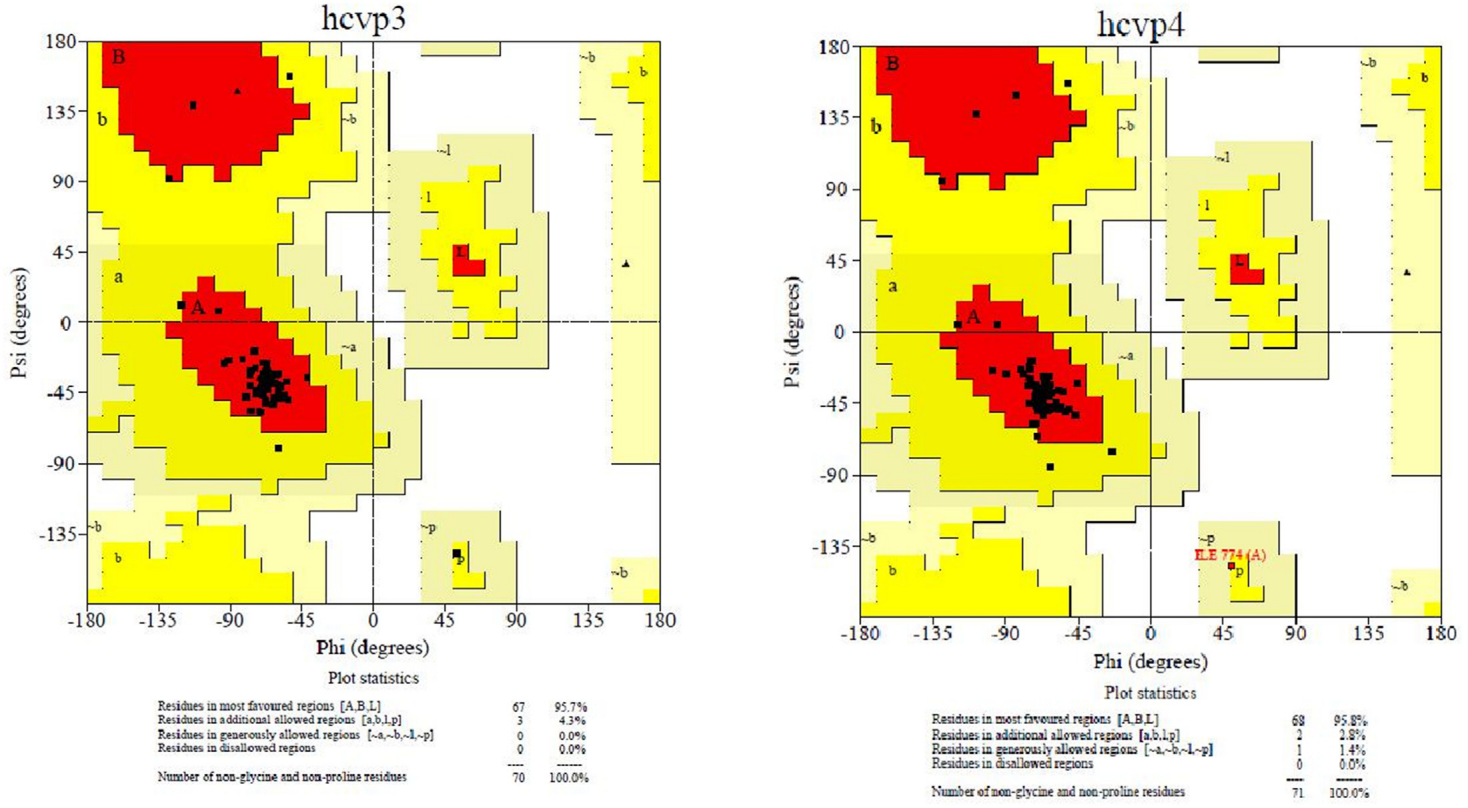
Ramachandran plot of model p7 from GT3 and GT4 plotted between pi and psi angles of all amino acids. Areas defined as red, yellow, light yellow and white indicate most favored, additional allowed, generously allowed and disallowed regions, respectively.

**Fig 4:**
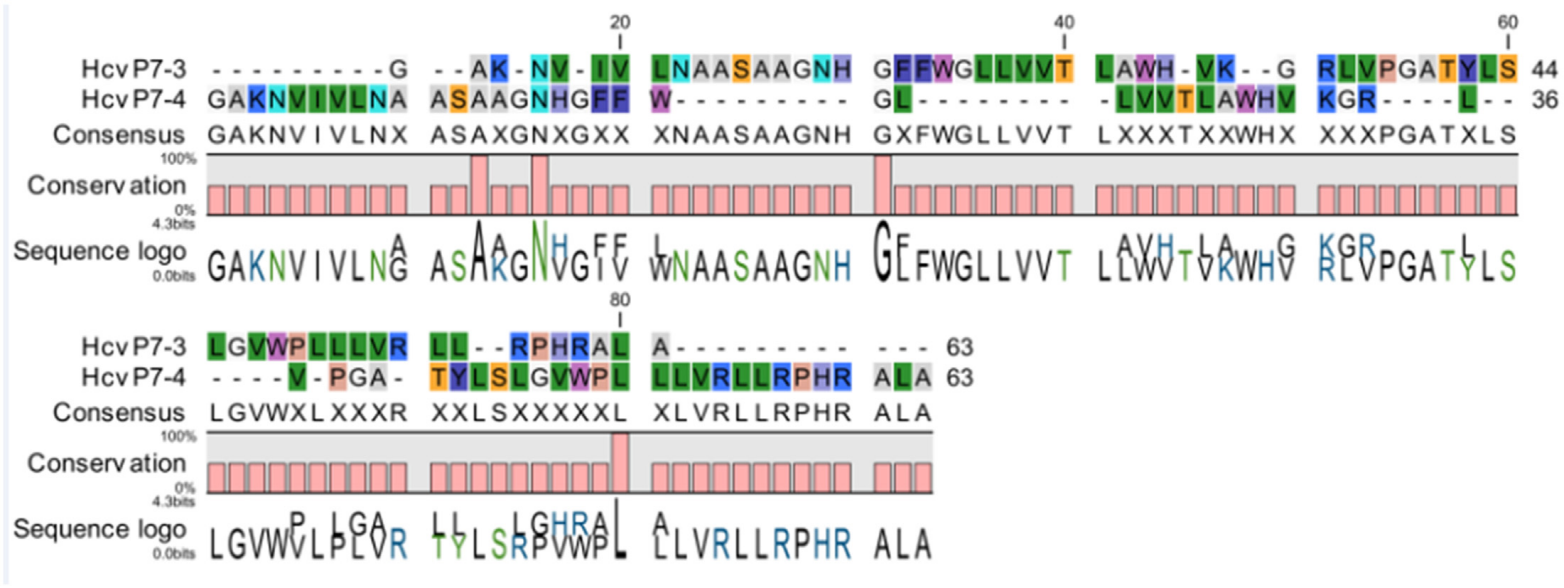
Modeled p7 alignment between GT3 and GT4 amino acid sequence and their conserved regions. The degrees of conservation as well as the consensus between the amino acid sequences have been also mentioned.

**Fig 5:**
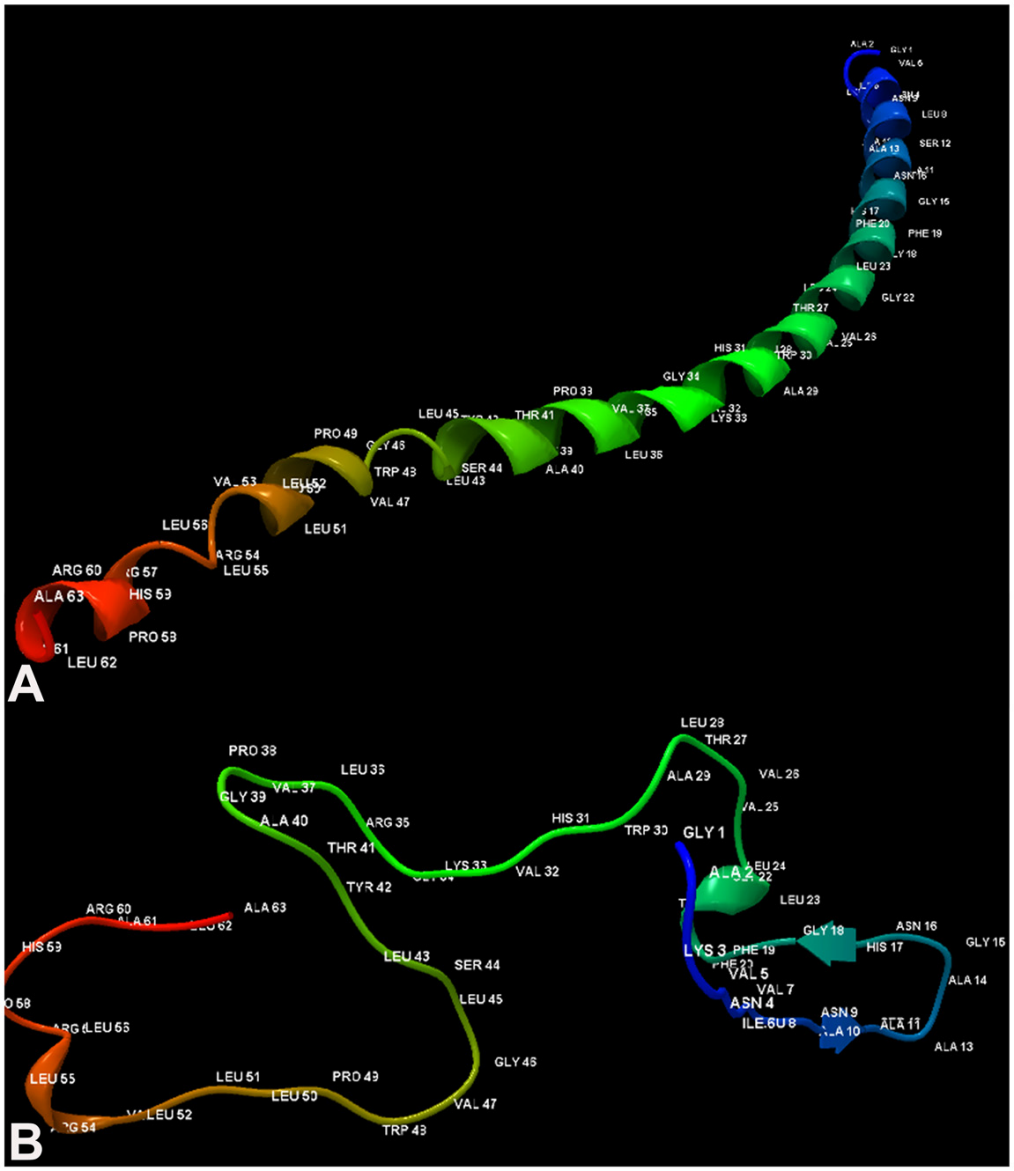
The 3D structure of both the modeled GT3 p7 and GT4 p7. (A) Coiled ribbon like structure of 63 amino acids p7 GT3 (b) Thread like structure of modeled p7 from GT4.

Both the modeled p7 ion channel were evaluated using the PROCHECK tool for stereochemical quality. By using Ramachandran plot, it was determined that both models had approximately 95% of AAs in the favored region, with less than 5% of the residues in the allowed region and 0.0% of the residues in the disallowed region (Table 3), indicating that the predicted models are highly reliable for further computational studies (Fig 3). Modeled p7 sequence alignment within GT3 and GT4 is denoted in Fig 4 with the consensus conserved regions. Fig 5 denotes the three-dimensional structure of both the GT3 and GT4 types p7 protein models. The RMSD value was calculated between the main-chain atom of the model and template, indicating close homology and ensuring reliability of the p7 model [61].

**Table 3:** Ramachandran plot describing the total number of residues as well as the number of residues located in allowed and disallowed regions for both the modeled p7 protein from HCV GT3 and HCV GT4.

Protein Models	Favored regions	Allowed region	Generously Allowed region	Disallowed region	Total number of residues
HCV-3-p7 model	95.7%	4.3%	0.0%	0.0%	80
HCV-4-p7 model	95.8%	2.8%	1.4%	0.0%	80
1ZDO	99.0%	0.8%	0.0%	0.0%	505

### QSAR analysis

VEGA-QSAR analysis was carried out to predict different biochemical properties of potential ligands. Results attained through QSAR models could be effective to evaluate the chemical properties of chosen compounds, decreasing the necessity of animal tests. Different models were tested against antiviral compounds (Tables 4, 5 and 6). The selected compounds had both positive and negative predictions, including both mutagenicity and carcinogenicity. The fathead minnow LC50 was predicted to be less than 6.0 [-log (mol/L)] for all selected compounds, except for that of EGCG and honokiol, which were about 6.4 [-log (mol/L)] and 6.0 [-log (mol/L)], respectively. All compounds are sensitive to the skin except EGCG; NB-DNJ is known to be non-toxic compared to the other selected ligands. Only apigenin and luteolin are biodegradable. All the selected compounds are non-carcinogenic except for naringenin, silymarin, and quercetin. QSAR models predict hexamethylene chloride, NB-DNJ, and BIT225 as mutagenic. The log *P* value is a valuable parameter to understand the behavior of drug molecules; log *P* value is higher in honokiol (5.58 log units) and nobiletin (3.99 log units). Apigenin, NB-DNJ, and BIT225 have log *P* values less than 1.50 log units.

**Table 4:** Predicted values for the selected drugs using VEGA QSAR and their applicability domain analysis for various models.

QSAR Models	Amantadine	Hexamethylene Chloride	NB-DNJ	BIT225
Fathead minnow LC50 (96hr)-log (mol/l)	2.97	2.13	2.06	4.08
Daphnia Magna LC50 (48hr)-log (mol/l)	4.75	4.63	3.48	4.98
Mutagenicity model (CAESAR)	Non-mutagen	Mutagen	Mutagen	Mutagen
Carcinogenicity model	Non-carcinogen	NON-Carcinogen	NON-Carcinogen	NON-Carcinogen
Developmental Toxicity model	Toxicant	Toxicant	Non- Toxicant	Toxicant
BCF modellog(l/kg)	1.48	2.02	0.07	0.67
Ready biodegradability model	NON Ready Biodegradable	NON Ready Biodegradable	NON Ready Biodegradable	NON Ready Biodegradable
LogP prediction[log units]	2.44	2.36	0.38	1.85
Skin sensitization model (CAESAR)	Sensitizer	Sensitizer	Sensitizer	Sensitizer
BCF read-acrosslog(l/kg)	2.17	1.86	0.22	1.50

**Table 5:** QSAR predicted values and their applicability domain analysis for various models for active flavonoids.

QSAR Models	Apigenin	EGCG	Ladanein	Luteolin	Naringenin	Silymarin	Quercetin
Fathead minnow LC50 (96hr)-log (mol/l)	4.42	6.42	4.95	4.95	4.58	4.58	4.58
Daphnia Magna LC50 (48hr)-log (mol/l)	2.61	1.76	4.46	3.25	2.61	2.61	2.61
Mutagenicity model (CAESAR)	NON-Mutagen	NON-Mutagen	NON-Mutagen	NON-Mutagen	NON-Mutagen	NON-Mutagen	NON-Mutagen
Carcinogenicity model	NON-Carcinogen	NON-Carcinogen	NON-Carcinogen	NON-Carcinogen	Carcinogen	Carcinogen	Carcinogen
Developmental Toxicity model	Toxicant	Toxicant	Toxicant	Toxicant	Toxicant	Toxicant	Toxicant
BCF modellog(l/kg)	0.53	0.01	0.53	0.53	0.53	0.53	0.53
Ready biodegradability model	Ready Biodegradable	Non- Ready Biodegradable	Ready Biodegradable	Ready Biodegradable	Non- Ready Biodegradable	Non- Ready Biodegradable	Non- Ready Biodegradable
LogP prediction[log units]	1.81	1.71	2.71	2.71	2.52	2.52	2.52
Skin sensitization model (CAESAR)	Sensitizer	Non- Sensitizer	Sensitizer	Sensitizer	Sensitizer	Sensitizer	Sensitizer
BCF read-acrosslog(l/kg)	1.26	0.97	2.25	2.25	2.18	2.18	2.18

**Table 6:** QSAR predicted values for phenol compounds used for docking analysis and their applicability domain analysis for various models.

QSAR Models	Honokiol	Nobelitin
Fathead minnow LC50 (96hr)-log (mol/l)	6	5.37
Daphnia Magna LC50 (48hr)-log (mol/l)	3.62	4.45
Mutagenicity model (CAESAR)	Non-mutagen	Non-mutagen
Carcinogenicity model	carcinogen	Non- carcinogen
Developmental Toxicity model	Toxicant	Toxicant
BCF modellog(l/kg)	2.06	0.39
Ready biodegradability model	Not-assignable	Ready Biodegradable
LogP prediction[log units]	5.58	3.99
Skin sensitization model (CAESAR)	sensitizer	Non- sensitizer
BCF read-acrosslog(l/kg)	2.33	1.95

### Virtual screening

#### Docking with selected drugs

**Fig 6:**
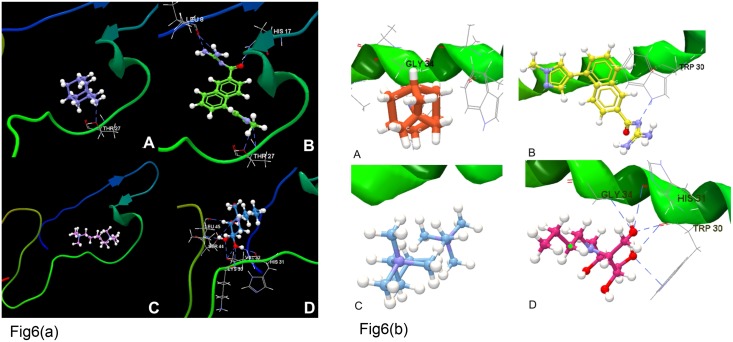
Computational docking study of GT3 p7 model and selected drugsMolecular binding of a set of selected ligands against GT3 p7 model (a) Amantadine, (b) BIT225, (c) Hexamethylene chloride, and (d) N-butyl-deoxynojirimycin. The Hydrogen bonds have been shown as blue dashed lines. Molecular interactions of GT4 p7 model (shown as green ribbon) with different ligands such as (a) Amantadine, (b) BIT225, (c) Hexamethylene chloride, and (d) N-butyl-deoxynojirimycin. Hydrogen bonds have been shown as blue dashed lines.

In the CLC Molecule Project, in an entry, docking results are displayed together with the protein and other molecules in the project to visualize the binding mode of the ligand in the binding site. The ‘create interacting atoms group’ option was used to generate a custom atom group consisting of protein residues and molecules having at least one heavy atom within 5 Å of a ligand heavy atom. HCV p7 is distinguished into three regions, the loop region includes residues from 25 to 45 and the terminal regions include residues from 1–13 and 50–63, respectively for N- and C- terminal sites. The binding affinities, along with the re-rank score, were calculated for the best complexes. NB-DNJ formed maximum H-bonds with both GT3 and GT4 and exhibited the highest binding affinities (-28.74 kcal/mol and -28.70 kcal/mol, respectively). NB-DNJ is the only ligand from selected drugs which exhibits larger number of interactions with p7 residues. NB-DNJ is capable of forming hydrogen bonds, as it has large aliphatic chains thereby having largest number of rotatable bonds. GT3 p7 and GT4 p7 protein residues formed only one H-bond at the Thr27 and Gly34 residues with carbon atoms of amantadine. Hexamethylene chloride did not have any interactions with the modeled p7 residues in either genotype. In GT3 p7, BIT225 formed the best complex, forming five H-bonds with Leu8 (2), His17, and Thr27 (2) with an interaction energy value of -38.38 kcal/mol; in GT4 p7, only one H-bond interaction formed at Trp30 (energy value -30.47 kcal/mol). Only BIT225 was observed to have interactions with the N-terminal region of GT3 p7 involving residues Leu8 (2) and His17 (binding energy value-38.38 kcal/mol). The highest interaction energy value obtained was -45.80 kcal/mol, formed by hexamethylene chloride, due to a larger number of H-bonds formed within the carbonyl backbone and its flexible side chains. The best interaction observed by docking studies of NB-DNJ was with GT4 p7 residues Gly34, His31 (2), and Trp30 (2). Comparing both p7 genotype models, only amantadine extended one H-bond interaction with p7 ion channel at Thr27 in GT3 and at Gly34 in GT4 (Fig 6a & 6b). These docking results indicate that both the C-terminal and N-terminal side regions of p7 contain potential drug interacting sites (Table 7). The known antiviral drugs such as amantadine may have less binding affinity compared to BIT225 and NB-DNJ, however, the difference is not very significant in both HCV p7 GT3 and GT4. Leon et al have also reported that amantadine exhibits weaker binding energies due to its interactions with the loop regions of the p7 protein of HCV GT1a [62]. Based on the interaction energy and residues forming H-bonds, the compounds were ranked in the following descending order with respect to predicted effectiveness of binding NB-DNJ > BIT225 > amantadine > hexamethylene chloride. The docking experiment conducted by in silico method is shown in S1 and S2 Video files.

**Table 7:** Molecular docking study between selected drugs along with both model of p7 GT3 and p7 GT4 and their intermolecular docking values presented with their interaction energy, HBond energy, docking score, number of interactions and the interacting residues.

P7 Domain	Derivative Name	Interacti on Energy (kcal/mol)	HBond Energy (kcal/mol)	Docking Score (kcal/mol)	Interactions	Protein Interactions
HCV-p7 GT3	Amantadine	-32.09	-2.4965	-35.963	1	Thr27
	Hexamethylenee chloride	-45.80	-2.4628	-68.981	0	-
	BIT225	-38.38	-4.1862	-43.737	5	Leu8(2),His17,Thr27(2)
	NB-DNJ	-28.74	-6.7858	-57.711	8	Leu45(2),Ser44(2),Lys33(2),Val32,His31
HCV-p7 GT4	Amantadine	-32.51	-2.5501	-23.413	1	Gly34
	Hexamethylenee chloride	-40.13	-2.9394	-65.746	0	-
	BIT225	-30.47	-6.2556	-47.140	1	Trp30
	NB-DNJ	-28.708	-8.0267	-63.972	5	Gly34,His31(2),Trp30(2)

#### Possible binding sites for flavonoids compounds

**Fig 7:**
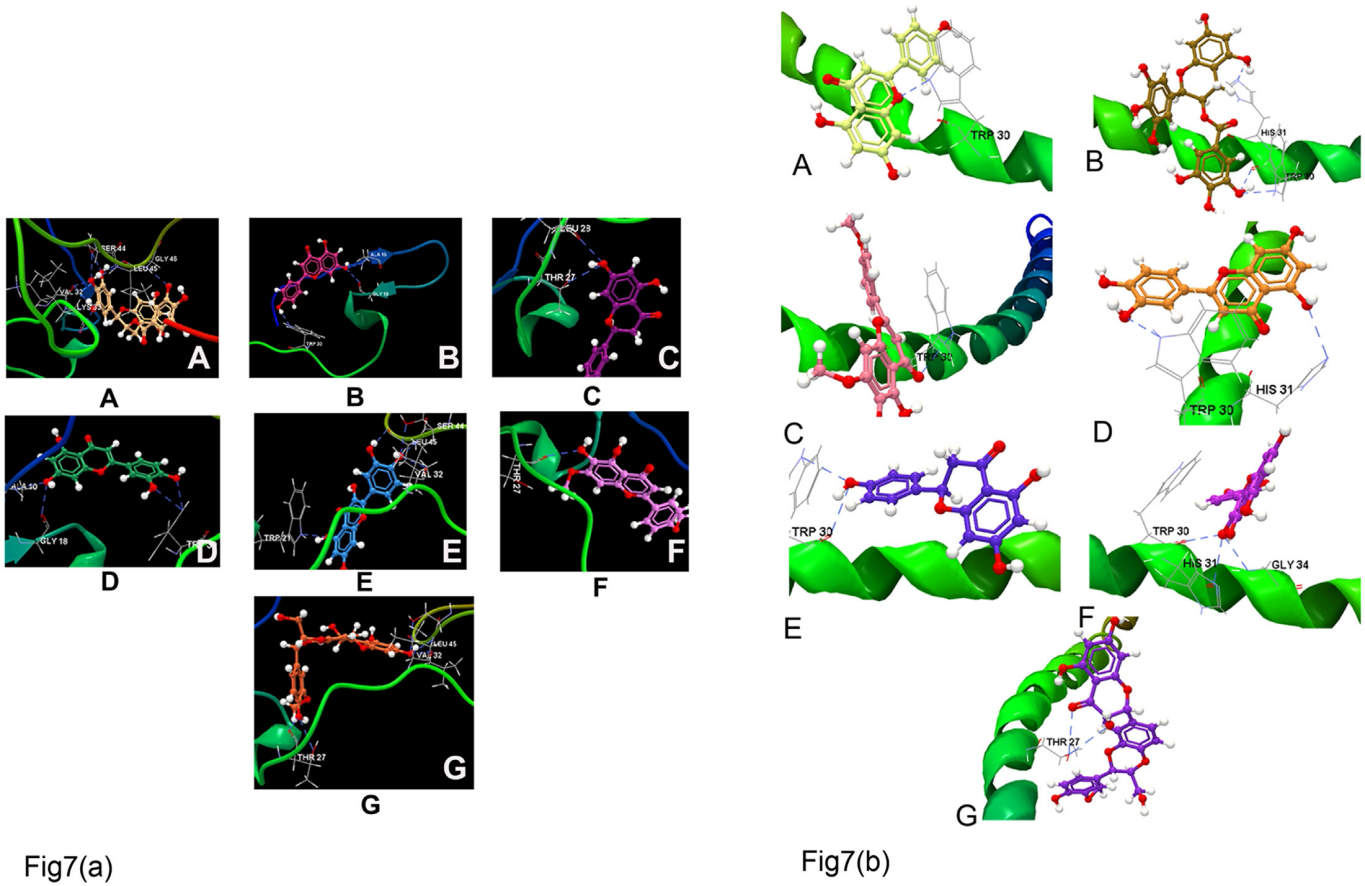
*In silico* docking study of p7 model of GT3 and GT4 with flavonoids. **(A)** Snapshots of molecular interactions of GT3 p7 model with different flavonoids such as (a) Epigallocatechin-3-gallate, (b) Apigenin, (c) Naringenin, (d) Luteolin, (e) Quercetin, (f) Ladanein, and (g) Silymarin. Hydrogen bonds have been shown as blue dashed lines.**(B)** Flavonoid interactions with the GT4 p7 model (shown as green ribbon). (a) Apigenin, (b) Epigallocatechin-3-gallate, (c) Ladanein, (d) Luteolin, (e) Naringenin, (f) Quercetin, and (g) Silymarin. Hydrogen bonds have been shown as blue dashed lines.

Using both the p7 ion channel models from GT3 and GT4, we show the interactions of both flavonoids and phenols involved in inhibition. Two main factors are critical to the success of a ligand-protein docking study. First, the energy function of binding to the proteins and second, the number of hydrogen bonds formed in the binding mode. In the GT3 p7 model, EGCG had the lowest binding energy due to binding interactions with Ser44 (2), Leu45 (2), Gly46, Val32, and Lys33 (2), forming as many as 8 H-bonds. It is, however, worth mentioning that EGCG has no effects on HCV RNA replication and on assembly or release of progeny virions [63, 64]. Therefore, this strong binding of EGCG may be assumed to inhibit the cell to cell spread of the virus to block the ion channeling process of p7 protein thereby disrupting the initial step of HCV cell entry [64]. The quercetin formed 5 H-bonds with Trp21, Val32, Leu45 (2), and Ser44 (Table 8). Luteolin and silymarin both formed four H-bonds. Only apigenin and luteolin interacted with the residues in the N-terminal site of GT3 p7, forming H-bonds with protein residues Ala10 and Gly18. Ladanein and naringenin exhibited high binding energy values and few interactions with the modeled HCV p7 protein. In analyzing the energy values of the HCV GT4 p7 model, the binding conformation score was much higher compared to that of the HCV GT3 p7 model. However, the number of interactions formed by natural molecules with HCV GT3 p7 model is significantly higher than the binding modes of drug interactions with HCV GT4 (Fig 7a and 7b). Most of the interactions formed by natural molecules targeted binding in the loop region which is essential for the mechanism and function of HCV p7.

**Table 8:** Selected bioactive flavonoids and their docking score along with the number of HBond formation with modeled p7 ion channel from both genotypes as obtained from molecular docking using CLC drug discovery analysis.

FlavonoidCompounds	HCV p7 GT 3			HCV p7 GT4		
	Docking Score(kcal/mol)	No of H bonds	Interacting proteins	Docking Score(kcal/mol)	No of H bonds	Interacting proteins
Apigenin	-48.23	3	Ala10, Gly18, Trp30	-58.35	1	Trp30
EGCG	-25.36	8	Ser44(2), Leu45(2), Gly46, Val32, Lys33(2)	-49.36	2	His31,Trp30
Ladanein	-60.25	1	Thr27	-70.82	1	Trp30
Luteolin	-35.26	4	Ala10, Gly18, Trp(30)	-37.36	2	Trp30,His31
Naringenin	-58.62	2	Leu28, Thr27	-52.41	1	Trp30
Quercetin	-35.56	5	Trp21, Val32, Leu45(2), Ser44	-45.81	3	Trp30,His31,Gly34
Silymarin	-38.26	4	Thr27(2), Val32, Leu45(2)	-50.04	1	Thr2

#### Possible binding sites for Phenol compounds

**Fig 8:**
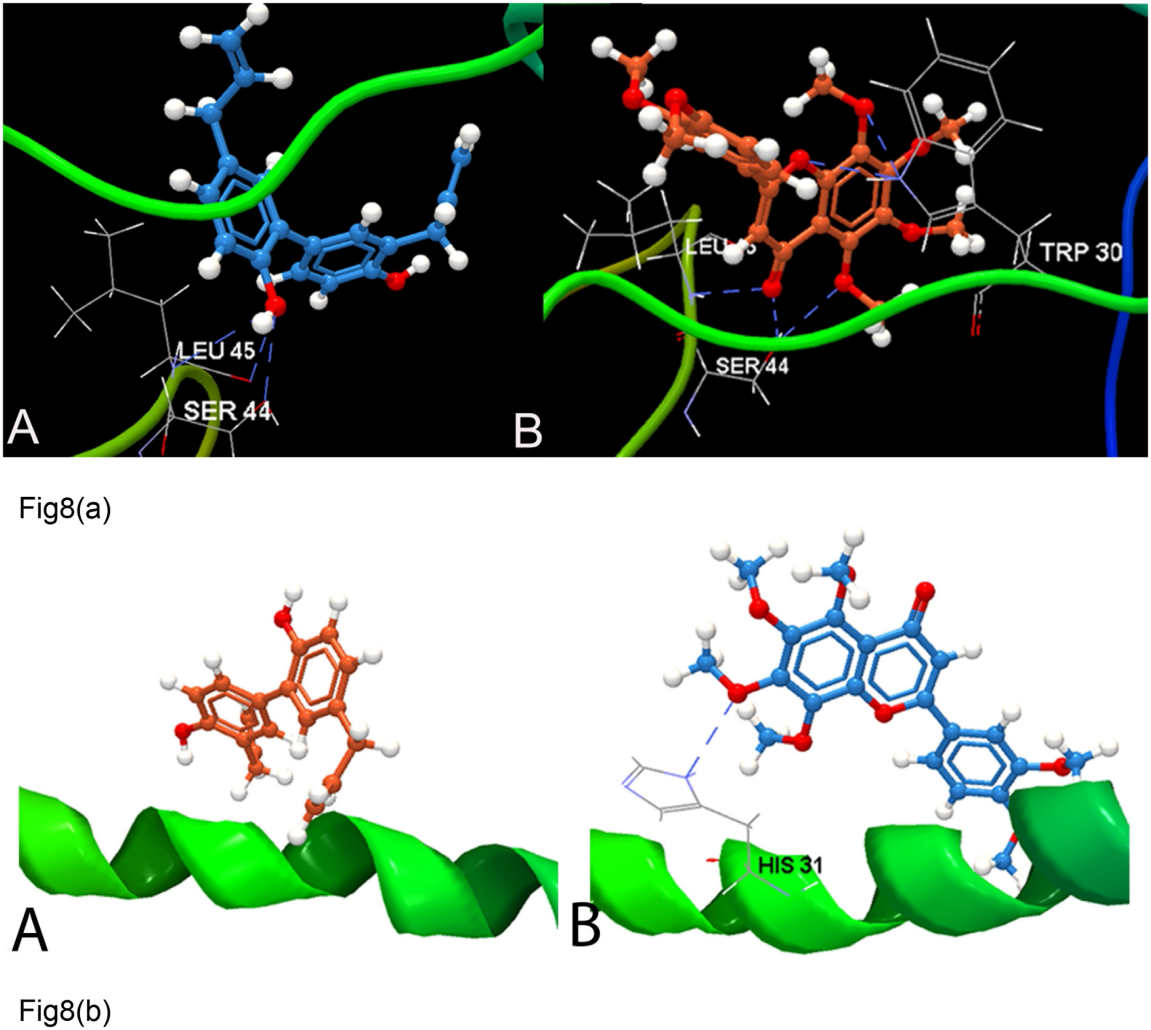
Analyzing docking interaction of p7 model between GT3 and GT4 with phenol compounds. **(A)** Molecular interaction of GT3 p7 model with phenolic compounds (a) Honokiol and its methoxy derivative (b) Nobiletin. Hydrogen bonds have been shown as blue dashed lines. **(B)** Interaction of GT4 p7 model (shown as green ribbon) with phenolic compounds (a) Honokiol (b) Nobiletin. Hydrogen bonds have been shown as blue dashed lines.

To analyze the reliability of interaction mode by looking at the energy function score with phenolic compounds within the HCV GT3 p7, the docking score is lower for honokiol which formed 3 HBonds with Leu45, Ser44 (2) and Trp30 (2) (shown in Table 9), whereas nobiletin formed a higher docking score of -48.32 kcal/mol forming interactions with Leu45 (2), Trp30 and Ser44 (2). No interaction was observed at the N- and C-terminal regions of the GT3 p7 and GT4 p7 models in case of phenol compounds. Notably, in HCV GT4 p7, a decrease in the binding energy values with very few HBond interactions was observed (Fig 8a and 8b).

**Table 9:** Phenol compounds and their interactions with modeled p7 proteins from both genotypes as obtained from molecular docking studies.

PhenolCompounds	HCV p7 GT3			HCV p7 GT4		
	Docking Score(kcal/mol)	No of H bonds	Interacting proteins	Docking Score(kcal/mol)	No of H bonds	Interacting proteins
HonokiolNobelitin	-36.25	3	Leu45,Ser44(2),Trp30(2)	-73.84	0	-
	-48.32	5	Leu45(2),Trp30,Ser44(2)	-60.31	1	His31

### Current p7-based antiviral strategies

p7 plays a vital role in viral assembly and discharge of mature viral particles and is, thus, highly conserved across HCV genotypes, making p7 an excellent potential latent antiviral drug target. Amantadine, long alkyl chain immunosugar derivatives, and hexamethylene amiloride have been established as channel-blocking compounds and it is shown that the p7 protein interacts with the non-structural protein 2 of the HCV present at the endoplasmic reticulum; this interface is crucial for the infectivity of the virus [65]. Some p7 inhibitors exhibiting antiviral activity in cell culture have been reported, largely from various experiments studied with viroporins from various other viruses. These inhibitors include amantadine, known to inhibit the influenza A virus M2 channel [66–69], hexamethylene amiloride, known to inhibit HIV-1 vpu ion channel [66], and long-alkyl-chain iminosugar derivatives [37].

#### Amantadine

Amantadine is chemically known as 1-adamantanamine hydrochloride which has a dual pharmacological action of treating viral and Parkinson disease. Its mechanism of action is mainly to inhibit the release of viral DNA into the host cells by interacting with the function of transmembrane domain of M2 protein. With respect to high prevalence rate of drug-resistant virus, the consumption of amantadine derivative like rimantidine for treating prophylaxis is not proposed in the country like USA [70]. Smith and co-workers studied the effectiveness of the treatment using amantadine in patients affected with HCV who were earlier known to have been failed to respond with the interferon therapy [71]. However, studies have failed to approve the positive effect of both the interferon therapy as well as with IFN-α/ribavirin [72, 73]. There is still a hopes for amantadine as it was observed to inhibits p7 function in artificial membranes [39] as well as in cell-based assay, that it in turn inhibit activity of viral hepatitis [74]. Griffin et al. validated various p7 inhibitor molecules against both HCV cell lines and in vitro assay in a parallel approach. They identified inhibition of viral entry and few compounds denoted antiviral activity specific to block the function of p7 ion channel [75].

#### Amiloride

Amiloride categorized itself in guanidium group of compounds containing pyrazine derivative. It functions by blocking the sodium channel present in the epithelial tissue thereby rendering sodium reabsorption in kidney, resulting in depletion of sodium from the body without losing potassium. The Vpu-protein of HIV-1 is similar to p7 forms cation channels in vitro and improves the budding thereby releasing virus infectious particles [76]. Ewart et al. and team discussed about the HIV-1 vpu-protein has similar property to p7 which releases viral particles during budding and the study validated that the derivatives of amiloride retard the activity of ion channel resulting in budding triggered by HIV-1 Vpu [66]. Recently hexamethylene amiloride, a derivative of amiloride is known for its inhibition activity against p7 ion channel [31]. The study of cell toxicity with varying drug concentrations is required in cell culture, to attain p7 inhibition precludes a strong decision regarding the inhibitory influence of amiloride on infectious virus particle synthesized from tissue culture system [75].

#### Iminosugars derivatives

Iminosugars deoxynojirimycin (DNJ) are monosaccharide sugar molecules in which the oxygen ring substituted by a nitrogen atom [77]. Glucose-derivatives (DNJ), such as N-nonyl-DNJ, and N-butyl-DNJ are effective inhibitors against ER α-glucosidases both I and II when experimented in HCV surrogate model [78]. The α-glucosidases are well-known to eliminate glucose molecules from the N-linked glycans present in high manose bonded and hence this processing step is vital for the further interaction between both ER chaperones and the glycoproteins [79]. Thus, compounds comprising of a DNJ header group along with long alkyl side chain are known to have dual roles inhibiting the activity of ER α-glucosidases and becoming a barrier for p7 channel function. Due to non-responders to IFN-α-based therapy, a new derived compound called NN-DNJ (UT-231B) has entered the clinical phase II study but the antiviral efficacy is not yet confirmed [80].

#### BIT225

A latest experimental drug developed from Biotron Limited for treating both HCV and HIV infection [81]. Moreover, BIT225 was capable to block the Vpu ion channel function by disrupting the HIV assembly with the host white blood cells. It also expressed antiviral synergy with NS5B polymerase inhibitors, ribavirin and IFN-α. The drug has been credited targeting p7 ion channel activity and has efficiently accomplished a phase Ia, in healthy volunteers with single dose trial and phase Ib to assess the pharmacokinetics of frequent medication for certain doses in HCV affected patients [81].

## Conclusions

In our study, the three dimensional structure of the p7 ion channel was modeled by using precise computational tools. The p7 ion channel protein from HCV was modeled in the absence of any complete p7 structure and hence high-resolution template structure was used. Studies have reported regarding various drug binding site that stabilize the closed p7 channels through an allosteric mechanism as proposed in controversial studies of IAV M2 [82–84]. It is still assumed that the p7 folds is characterized as a hairpin as it still remains indistinct regarding rearrangements of the protein structure between the monomer and hexamer forms [80, 82]. Fascinatingly, an amiloride-based GT1a p7 inhibitor and BIT225 [80], is presently under clinical trials combined with ribavirin and interferon. We conclude the depth of the number of residues binding modes and the native binding energy is obscured with the change in the protein structure of the two genotypes. In this work, molecular docking has been performed with 10 naturally occurring plant extracts and four known drugs to inhibit p7 activity. The loop region of the p7 has been found to harbor residues necessary for the mechanism of function of p7. It is also hypothesized that binding of the ligand molecules in the loop region inactivates essential dynamics required for the protein. Drugs have variable levels of interactions from GT to GT. Antiviral drugs, whose effectiveness is limited to GT1 may not be as effective in GT3 and GT4. Currently, there is no one-size-fits-all treatment available. Sequence variation in genotype validates and determines the specificity from GT to GT as well as from sequence to sequence. In light of our reported data set, our present *in silico* study supports the sequence variation which determines the drug interaction and enhances the benefit of multiple DAA combinations.

## Perspectives on the Future Directions

This computational study will show an impulse to start broader screening for small natural molecules as HCV p7 inhibitors. The key molecules screened and analyzed in this study should be a promising starter for large scale screening from the list of large number of chemical compounds available from the Ligand info meta database by using the latest virtual screening methods. Current advancement in understanding the molecular basis for p7 function might also shoot interest in designing and developing compounds that can target key residues. Continuous research and recent advances in the field of science will hopefully pay result in the discovery of more natural compounds for use in the laboratory and clinical trials with lesser side effects. Deeper biochemical knowledge of the complex p7 nature will in due course help to describe the molecular mechanisms of ion channel and its folding which can be a vital to achieve an efficient drug against human and animal virus particles. In summary, we aligned the entire p7 structure from all genotypes available, modeled the p7 protein from GT3 (Asia) and GT4 (Middle East) and compared its docking interaction with both known inhibitors as well natural compounds that are known to have antiviral properties. This study will build a way to research in depth the molecular mechanism of interactions of p7, of other HCV genotypes and support for screening more specific natural inhibitors from available chemical and biological medicinal plant extracts.

## Supporting Information

S1 Dataset Genotype sequence and multiple alignment. (DOC)

S1 Video *In silico* docking study of p7GT3. (MP4)

S2 Video Computational docking study of p7GT4. (MP4)
