# Supplementary material for: Computational Docking Study of p7 Ion Channel from HCV Genotype 3 and Genotype 4 and Its Interaction with Natural Compounds
Source: PLoS One. 2015 Jun 1;10(6):e0126510. doi: 10.1371/journal.pone.0126510 (PMC4451521; doi:10.1371/journal.pone.0126510)

1. **S1 Text: Genotype sequence and multiple alignment**

**P7 sequences from six genotypes**

**GT1**

**>isolateH-1a|P27958|747-809**

**ALENLVILNAASLAGTHGLVSFLVFFCFAWYLKGRWVPGAVYALYGMWPLLLLLLALPQRAYA**

**>isolate1-1a|P26664|747-809**

**ALENLVILNAASLAGTHGLVSFLVFFCFAWYLKGKWVPGAVYTFYGMWPLLLLLLALPQRAYA**

**>isolateHC-J1-1b|Q00269|747-809**

**ALENLVILNAASLAGTRGLVSFLVFFCFAWYLKGRWVPGAAYALYGMWPLLLLLLALPQRAYA**

**>isolateJapanese-1b|P26662|747-809**

**TLENLVVLNAASVAGAHGLLSFLVFFCAAWYIKGRLVPGAAYALYGVWPLLLLLLALPPRAYA**

**>isolateHCR6-1b|Q913V3|747-809**

**ALENLVVLNAASVAGAHGILSFLVFFCAAWYIKGKLVPGAAYAFYGVWPLLLLLLALPPRAYA**

**>isolateHC-J4-1b|O92972|747-809**

**ALENLVVLNAASVAGAHGILSFLVFFCAAWYIKGRLAPGAAYAFYGVWPLLLLLLALPPRAYA**

**>isolateBK-1b|P26663|747-809**

**ALENLVVLNSASVAGAHGILSFLVFFCAAWYIKGRLVPGATYALYGVWPLLLLLLALPPRAYA**

**>isolateTaiwan-1b|P29846|747-809**

**ALENLVVFNAASVAGMHGTLSFLVFFCAAWYIKGRLVPGAAYALYGVWPLLLLLLALPPRAYA**

**>isolateHC-G9-1c|Q81754|747-809**

**ALENLIVLNAASLVGTHGIVPFFIFFCAAWYLKGKWAPGLAYSVYGMWPLLLLLLALPQRAYA**

**>isolateIndia-1c|Q913D4|747-809**

**ALENLIVLNAASLAGTHGIVPFFIFFCAAWYLKGKWAPGLVYSVYGMWPLLLLLLALPQRAYA**

**GT2**

**>isolateHC-J6-2a|P26660|751-813**

**ALEKLVVLHAASAASCNGFLYFVIFFVAAWYIKGRVVPLATYSLTGLWSFGLLLLALPQQAYA**

**>isolateJFH-1-2a|Q99IB8|751-813**

**ALEKLVVLHAASAANCHGLLYFAIFFVAAWHIRGRVVPLTTYCLTGLWPFCLLLMALPRQAYA**

**>isolateHC-J8-2b|P26661|751-813**

**ALEKLIILHSASAASANGPLWFFIFFTAAWYLKGRVVPVATYSVLGLWSFLLLVLALPQQAYA**

**>isolate JPUT971017-2b|Q9DHD6|751-813**

**ALEKLIILHSASAASANGPLWFFIFFTAAWYLKGRVVPAATYSVLGLWSFLLLVLALPQQAYA**

**>isolateBEBE1-2c|Q68749|751-813**

**ALEKLVILHAASAASSNGLLYFILFFVAAWCIKGRAVPMVTYTLLGCWSFVLLLMALPHQAYA**

**>isolateVAT96-2k|Q9QAX1|751-813**

**ALEKLVILHAASAASSHGMLCFIIFFIAAWYIKGRVTPLVTYSYLGMWSFSLLLLALPQQAYA**

**GT3**

**>isolatek3a-3a|Q81495|753-815**

**ALENLVTLNAVAAAGTHGIGWYLVAFCAAWYVRGKLVPLVTYSLTGLWSLALLVLLLPQRAYA**

**>isolateNZL1-3a|Q81258|753-815**

**ALENLVTLNAVAAAGTHGIGWYLVAFCAAWYVRGKLVPLVTYSLTGLWSLALLVLLLPQRAYA**

**>isolateTr-Kj-3b|Q81487|755-817**

**AMENLVMLNALSAAGQQGYVWYLVAFCAAWHIRGKLVPLITYGLTGLWPLALLDLLLPQRAYA**

**>isolateJK049-3k|Q68801|752-814**

**ALENLIVLNAISAAGTHGIWWSLVAFCVAWHVRGRIFPIAVYSIVGLWPLLLLVLMLPYRAYA**

**GT4**

**>isolateED43-4a|O39929|747-809**

**ALSNLININAASAAGAQGFWYAILFICIVWHVKGRFPAAAAYAACGLWPCFLLLLMLPERAYA**

**>isolate**[**M1VKT9**](http://www.uniprot.org/uniprot/M1VKT9)**-4a|747-809**

**ALSNLININAASAAGTQSFWYAILFICIAWHVKGRLPAIAAYAACGMWPLLLLLLMLPERAYA**

**>isolate**[**A2CJ00**](http://www.uniprot.org/uniprot/A2CJ00)**-4d|747-809**

**LANLITINAVSVAGIHGFWHAILLICIAWHVKGRFPAAATYAACGLWPLLLLVLMLPERAYAF**

**>isolate**[**Q1ZZ56**](http://www.uniprot.org/uniprot/Q1ZZ56)**-4d|747-809**

**LANLVTINAVSAAGTHGFWYAILVICIAWHVKGRIPAAATYAACGMWPLLLLVLMLPERAYAF**

**>isolate**[**A0A023JCC8**](http://www.uniprot.org/uniprot/A0A023JCC8)**-4d|747-809**

**LANLITINAVSVASIHGFWYAIFVICIAWHVKGKLPAAATYAACGLWPLLLLVLMLPERAYAF**

**>isolate**[**A8S500**](http://www.uniprot.org/uniprot/A8S500)**-4f|747-809**

**EAALTNLININAAAAVGTHGFYYAILFICVVWYIKGRAPAAAAYAACGMWPLLLLLLALPERA**

**>isolate**[**A8S507**](http://www.uniprot.org/uniprot/A8S507)**-4f|747-809**

**AALANLITINATAAVGTHGFCYAILFICVVWYIKGRGPAAAAYAACGMWPLLLLLLALPERAY**

**GT5**

**>isolateEUH1480-5a|O39928|748-810**

**TCKNVIVLNAAAAAGNHGFFWGLLVVCLAWHVKGRLVPGATYLCLGVWPLLLVRLLRPHRALA**

**>isolateSA13-5a|O91936|748-810**

**ALENVIVLNAAAAAGTHGFFWGLLVICFAWHFKGRLVPGATYLCLGIWPLLLLLFLLPQRALA**

**GT6**

**>isolate6a33-6a|Q5I2N3|752-814**

**AVERLVVLNAASAAGTAGWWWAVLFLCCVWYVKGRLVPACTYMALGMWPLLLTILALPHRAYA**

**>isolateEUHK2-6a|O39927|751-813**

**AVERLVVLNAASAAGTAGWWWAVLFLCCVWYVKGRLVPACTYMALGMWPLLLTILALPPRAYA**

**>isolateTh580-6b|O92529|752-814**

**ALERLVVLNAASAAGTAGWCWTLIFLCCVWHVKGRLVPACTYTALGMWPILLVILALPQRAYA**

**>isolateVN235-6d|O92530|745-807**

**ALENLIVLNAASAASSQGWIYCLVFICCAWYIKGRVVPGATYAILHLWPLLLLVLALPQRAYA**

**>isolateVN004-6h|O92532|748-810**

**ALENVIVLNAASAASCQGLLWGLIFICCAWHVRGRAVPVTTYALLQLWPLLLLILALPRRAYA**

**>isolateJK046-6g|Q68798|751-813**

**ALENLIVLNAASAAATRGWECFLLFMCWAWYVRGRVVPAVTYGLLNLWPLLLLVLLLPHRAYA**

**>isolateVN405-6k|O92531|749-811**

**ALENLIVLNATSAAGSQGWVWGVVFICAAWYIRGRAAPITTYAILQLWPLLLLVLALPRRAYAALA**

**Multiple Sequence alignment between different genotypes and subtypes**


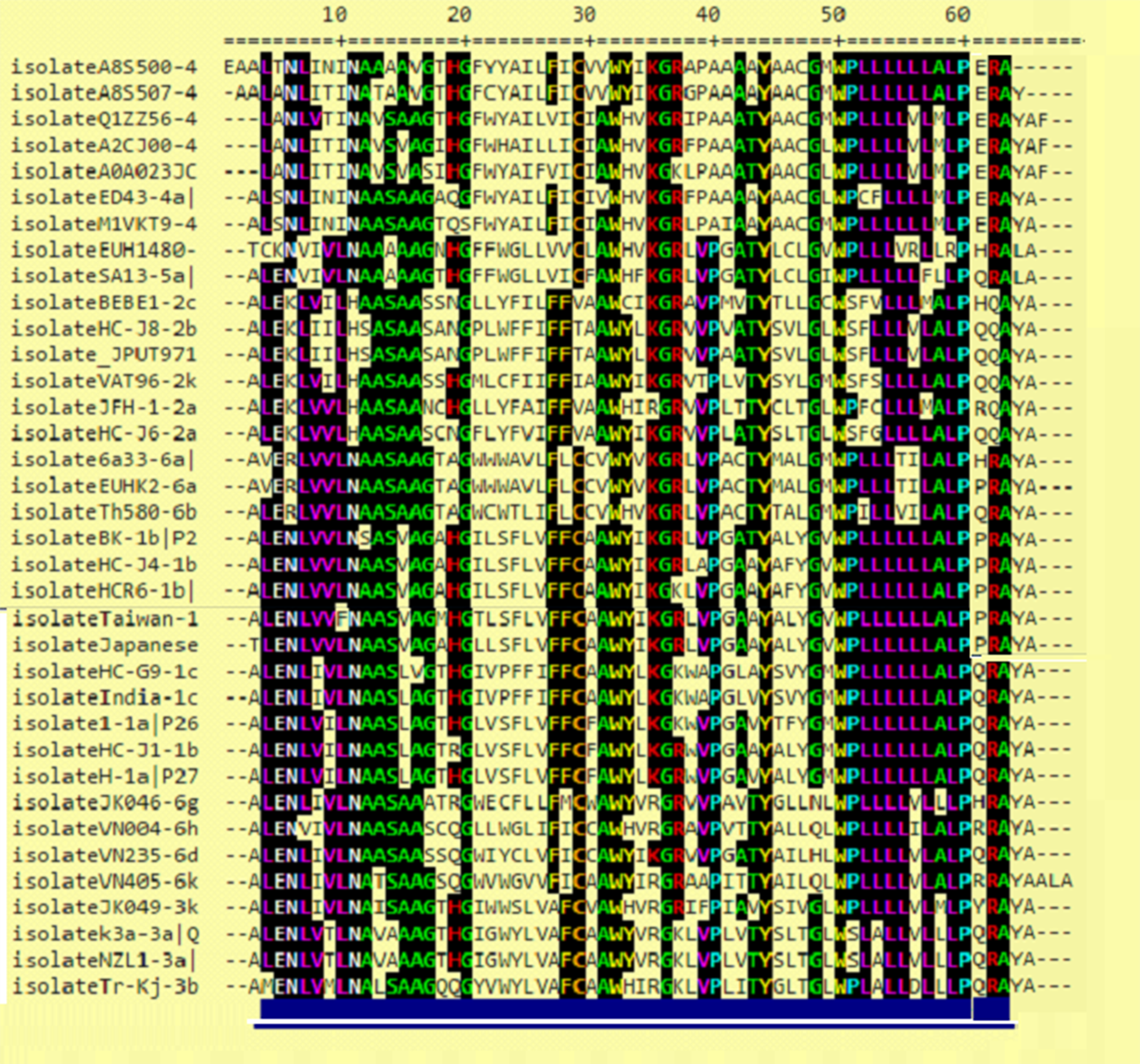


**Multiple sequence alignment between GT3 and GT4 subtypes**

**Genotype 3**

Sequence 1: isolatek3a-3a|Q81495|753-815 63 aa

Sequence 2: isolateNZL1-3a|Q81258|753-815 63 aa

Sequence 3: isolateTr-Kj-3b|Q81487|755-817 63 aa

Sequence 4: isolateJK049-3k|Q68801|752-814 63 aa

Sequences (1:2) Aligned. Score: 100

Sequences (1:3) Aligned. Score: 77.7778

Sequences (1:4) Aligned. Score: 68.254

Sequences (1:2) Aligned. Score: 100

Sequences (1:3) Aligned. Score: 77.7778

Sequences (1:4) Aligned. Score: 68.254

Sequences (2:3) Aligned. Score: 77.7778

Sequences (2:4) Aligned. Score: 68.254

Sequences (3:4) Aligned. Score: 61.9048

**Genotype 4**

Sequence 1: isolateED43-4a|O39929|747-809 63 aa

Sequence 2: isolateM1VKT9-4a|747-809 63 aa

Sequence 3: isolateA2CJ00-4d|747-809 63 aa

Sequence 4: isolateQ1ZZ56-4d|747-809 63 aa

Sequence 5: isolateA0A023JCC8-4d|747-809 63 aa

Sequence 6: isolateA8S500-4f|747-809 63 aa

Sequence 7: isolateA8S507-4f|747-809 63 aa

Sequences (1:2) Aligned. Score: 87.3016

Sequences (1:3) Aligned. Score: 77.7778

Sequences (1:4) Aligned. Score: 76.1905

Sequences (1:5) Aligned. Score: 73.0159

Sequences (1:6) Aligned. Score: 74.6032

Sequences (1:7) Aligned. Score: 73.0159

Sequences (1:2) Aligned. Score: 87.3016

Sequences (1:3) Aligned. Score: 77.7778

Sequences (1:4) Aligned. Score: 76.1905

Sequences (1:5) Aligned. Score: 73.0159

Sequences (1:6) Aligned. Score: 74.6032

Sequences (1:7) Aligned. Score: 73.0159

Sequences (2:3) Aligned. Score: 76.1905

Sequences (2:4) Aligned. Score: 80.9524

Sequences (2:5) Aligned. Score: 74.6032

Sequences (2:6) Aligned. Score: 76.1905

Sequences (2:7) Aligned. Score: 74.6032

Sequences (3:4) Aligned. Score: 88.8889

Sequences (3:5) Aligned. Score: 90.4762

Sequences (3:6) Aligned. Score: 65.0794

Sequences (3:7) Aligned. Score: 69.8413

Sequences (4:5) Aligned. Score: 87.3016

Sequences (4:6) Aligned. Score: 69.8413

Sequences (4:7) Aligned. Score: 74.6032

Sequences (5:6) Aligned. Score: 61.9048

Sequences (5:7) Aligned. Score: 66.6667

Sequences (6:7) Aligned. Score: 90.4762


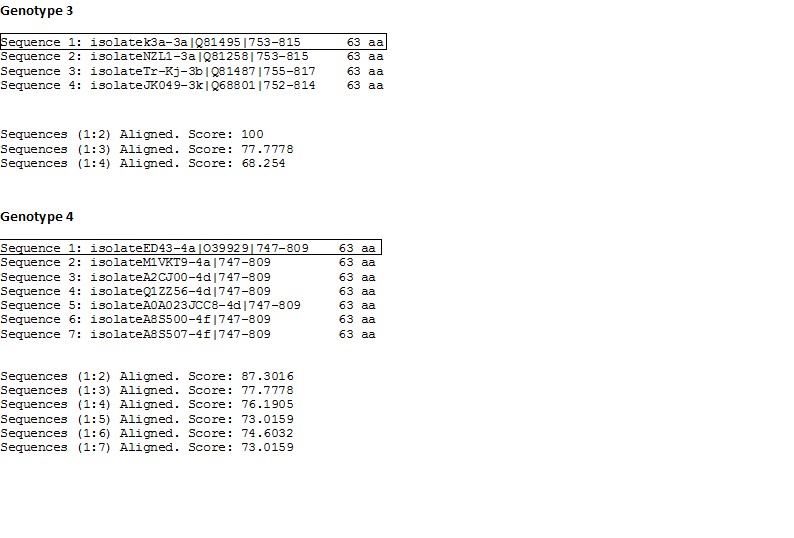

Supplement: S1 Dataset — (DOC) [file pone.0126510.s001.doc]
